# Perspectives on Atomic-Scale Switches for High-Frequency Applications Based on Nanomaterials

**DOI:** 10.3390/nano11030625

**Published:** 2021-03-03

**Authors:** Mircea Dragoman, Martino Aldrigo, Daniela Dragoman

**Affiliations:** 1 National Institute for Research and Development in Microtechnologies (IMT Bucharest), Erou Iancu Nicolae Street 126A, 077190 Voluntari, Romania; martino.aldrigo@imt.ro; 2 Physics Faculty, University of Bucharest, P.O. Box MG-11, 077125 Bucharest, Romania; daniela.dragoman@unibuc.ro; 3 Academy of Romanian Scientists, Splaiul Independentei 54, 050094 Bucharest, Romania

**Keywords:** microwaves, millimetre-waves, switches, memristor, ferroelectric, tunneling junction

## Abstract

Nanomaterials science is becoming the foundation stone of high-frequency applications. The downscaling of electronic devices and components allows shrinking chip’s dimensions at a more-than-Moore rate. Many theoretical limits and manufacturing constraints are yet to be taken into account. A promising path towards nanoelectronics is represented by atomic-scale materials. In this manuscript, we offer a perspective on a specific class of devices, namely switches designed and fabricated using two-dimensional or nanoscale materials, like graphene, molybdenum disulphide, hexagonal boron nitride and ultra-thin oxides for high-frequency applications. An overview is provided about three main types of microwave and millimeter-wave switch: filament memristors, nano-ionic memristors and ferroelectric junctions. The physical principles that govern each switch are presented, together with advantages and disadvantages. In the last part we focus on zirconium-doped hafnium oxide ferroelectrics (HfZrO) tunneling junctions (FTJ), which are likely to boost the research in the domain of atomic-scale materials applied in engineering sciences. Thanks to their Complementary Metal-Oxide Semiconductor (CMOS) compatibility and low-voltage tunability (among other unique physical properties), HfZrO compounds have the potential for large-scale applicability. As a practical case of study, we present a 10 GHz transceiver in which the switches are FTJs, which guarantee excellent isolation and ultra-fast switching time.

## 1. Introduction

The switch is a key device with multiple applications in nanoelectronics. A switch has two distinct states, i.e., ON and OFF, and these two states can be implemented in various ways. The electronic switch is a semiconductor component in which the carrier conduction is enabled (ON-state) or disabled (OFF-state) via a control parameter; the latter is the applied voltage in the case of two-terminal devices (such as diodes), or a gate voltage in the case of three-terminal devices. The digital electronics is based on these simple principles and a microprocessor contains billions of transistors integrated on a single chip, these transistors representing electronic switches dedicated to compute and memorize a huge quantity of information in a very short time. These digital switches have nano-sized dimensions, the ultimate target being their implementation with atomically thin materials, thus opening the path to electronics at atomic scale [1].

In the case of high-frequency signals, microwave or millimeter-wave switches are routing the signals between different ports, and hundreds or even thousands of them are used in a phased antenna array, in a transmission/reception system (e.g., radars) and in many high-quality items of equipment. In the ON-state, the high-frequency signals are transmitted between two different points of the same circuit, with a small insertion loss (i.e., less than 2 dB). By contrast, the OFF-state isolates two parts of a circuit and, in this case, the transmission must be very low i.e., the isolation must be between −25 and −40 dB. Semiconductor switches based on PIN diodes or field-effects transistors are used as microwave switches, but they are replaced nowadays by micro electro-mechanical systems (RF MEMS) having lower insertion loss, higher isolation and larger bandwidth [2]. In this type of switch, the ON- and OFF-state are created by the direct current (DC) attraction or repeal of a metallic membrane by a bottom electrode. However, RF MEMS have switching time of tens of microseconds (μs), and high actuation voltages in the order of tens of volts. Therefore, nano-electro-mechanical systems (NEMS) are used instead, since they show a low actuation voltage of few volts and switching time in the order of ns. In this frame, NEMS based on carbon nanotubes have emerged as microwave switches [3], but the fabrication is very difficult and the reproducibility is a challenge.

The question that arises from this brief review about microwave switches is whether microwave switches could attain the ultimate miniaturization conferred by atomically thin materials, as in the case of transistors. The answer is positive, as microwave atomic-scale switches are all based on memristors of different types, which are all non-volatile resistive switches, also exploiting atomically thin oxides with a thickness in the range 0.05–6 nm, but a lot of work remains regarding fabrication at the wafer scale and reproducibility.

## 2. Atomic-Scale Filament Memristor Microwave Switches

Atomic-scale or atomically thin materials are either van der Waals materials, also known as 2D materials (such as graphene and molybdenum disulphide MoS_2_), or hexagonal boron nitride (h-BN) which is made of billions of monolayers bound together by weak van der Waals forces in the vertical plane, while in the horizontal plane there are strong bonds between atoms. Graphene is a semi-metal, MoS_2_ is a semiconductor, and h-BN is an insulator, thus offering a large variety of atomic-thin materials, each of them with its own physical properties [4]. The majority of the atomic-scale switches are vertical devices such as MIM (metal–insulator–metal) or MSM (metal–semiconductor–metal) structures, where the insulator or semiconductor have atomically thin dimensions. If a MIM or MSM have the additional property of being a non-volatile resistive memory, then it is termed memristor. Moreover, the current-voltage must be pinched at 0 V. The known memristors are mostly based on oxides having a large density of oxygen vacancies [5].

**Figure 1:**
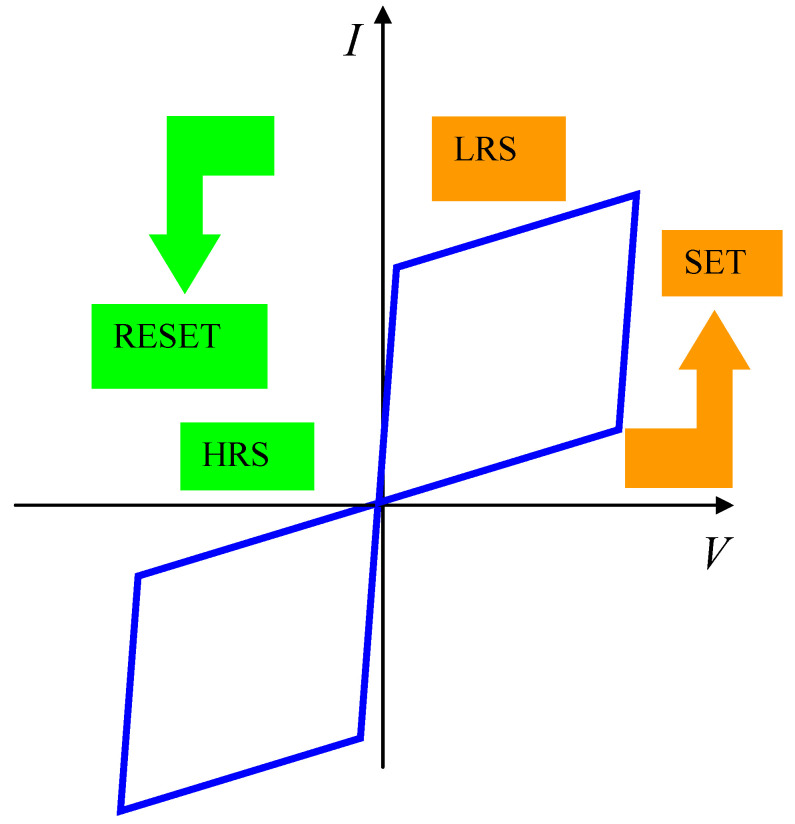
The schematic representation of a current-voltage dependence on a memristor.

In any memristor, the resistance R is reversibly changed from a high-resistance state (HRS), corresponding to the OFF-state (*R_OFF_*), to a low-resistance state (LRS), associated to the ON-state (*R_ON_*). The process of resistance change from HRS to LRS is termed as *Set*, while the inverse process (resistance change from LRS to HRS) is termed *Reset*. In bipolar memristors, the *Set* voltage signal is positive, thus transforming the atomically thin material from an insulation-like state (hence, displaying an HRS) to a metallic-like state (associated with an LRS). The process is controlled by a current compliance; otherwise, the memristor could be destroyed by high current values. The memristor remains in the LRS until a *Reset* negative voltage is applied to transform the memristor state from LRS to HRS. Therefore, any memristor behaves as a non-volatile memory. The current-voltage dependence of any memristor is represented in Figure 1.

Since during the reversible insulator-metal process the conductance of the memristor is modulated in time, i.e., it increases during the transition from HRS to LRS and decreases in the transition from HRS to LRS, any memristor can be seen as an artificial synapse [6] and, thus, many applications in the new field of neuromorphic computing can be envisaged. The equation of any memristor is given as follows:

*i*(*t*) = *v*(*t*)/{*R_ON_ γ*(*t*) + *R_OFF_* [1 − *γ*(*t*)]} (1)

where *γ*(*t*) is a continuous time-dependent function with values in the range [0, 1]. The function *γ*(*t*) reaches its maximum and minimum values, of 1 and 0, when *R* = *R_ON_* (LRS) and *R* = *R_OFF_* (HRS), respectively. *γ*(*t*) is considered as a linear function of the flux linkage.

A recent review about memristors and their applications as memories and synapses can be found in [1,7]. Furthermore, the majority of memristors are oxide memristors, in which the reversible transformation from HRS to LRS is due to conduction filaments formed inside the oxide; for this reason, they are also termed as filament memristors.

**Figure 2:**
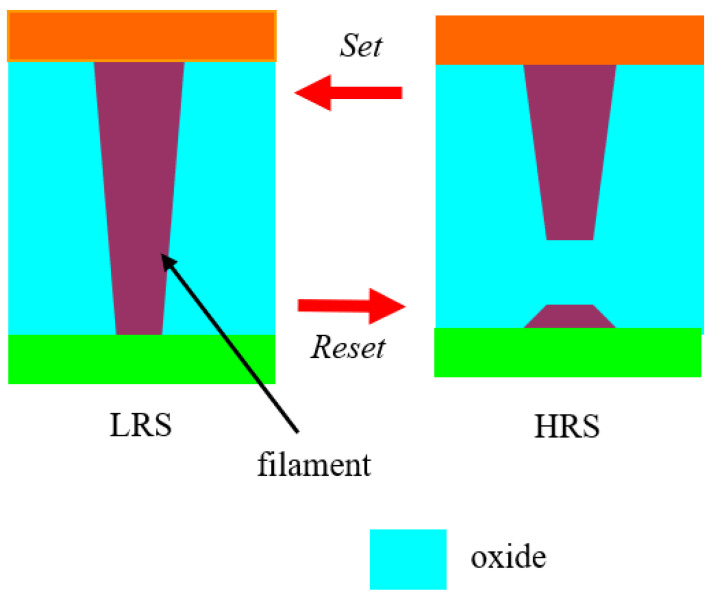
Schematic representation of the filament memristor and its states.

In the *Set* process, pairs of oxygen vacancies (anions) are created inside the oxide due to the impact ionization process: O_L_ → V_O_^2+^ + O_i_^2−^, where O_L_ are the oxygen atoms in the oxide lattice, V_O_^2+^ are the oxygen vacancies and O_i_^2−^ are supplementary oxygen ions produced in the lattice. The O_i_^2−^ ions are migrating to the top electrode, while the oxygen vacancies are migrating in the opposite direction to the bottom electrode, and trap the electrons. Thus, at the bottom electrode the population of oxygen vacancies is formed and is propagating to the top electrode, which causes the formation of tiny current filaments, hence providing conduction in the oxide and, as a consequence, the transition from an insulating to a conduction state (i.e., the transition from HRS to LRS). In the *Reset* process, the large densities of current filaments are cancelling oxygen vacancies via Joule heating: V_O_^2+^ + O_i_^2−^ → O_L_. Therefore, we have the inverse transition from LRS to HRS. These processes are depicted schematically in Figure 2.

**Figure 3:**
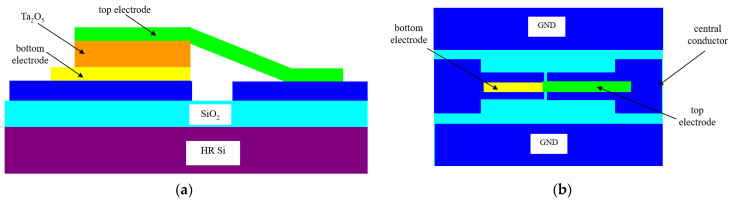
The microwave filament memristor in coplanar waveguide (CPW) technology: (**a**) cross-section; (**b**) top-view.

Soon after the discovery of the memristor, it was used as a high-frequency switch [8]. This was a logical step, since memristors have low *R_ON_* (in the range of 10–100 Ω), while *R_OFF_* is in order of tens or hundreds of MΩ. The oxide memristor is embedded in a planar waveguide termed coplanar waveguide (CPW), consisting in three metallic electrodes separated by two gaps, with the central conductor being the signal line and the outer electrodes being the ground planes; all the three electrodes are deposited on an insulator substrate. The CPW line has both input and output impedances of 50 Ω (standard reference impedance for microwave circuits). In the case of the microwave memristor switch described in [8], the substrate is a 525 μm-thick high-resistivity (HR, with resistivity of 1000 Ω·cm) silicon (Si) wafer having a 200 nm-thick silicon dioxide (SiO_2_) layer grown over it, such that the memristor is isolated from the substrate containing surface charges. The memristor is a MIM-like device, having two dissimilar metallic electrodes: the bottom electrode is Ti (5 nm)/Pt (20 nm), while the top electrode is Ta (30 nm)/Pt (200 nm). The oxide is amorphous Ta_2_O_5_ having the thickness of 7 nm, with a high density of oxygen vacancies. This memristor is integrated in the central electrode of the CPW as it is shown in Figure 3. The *Set* pulse has an amplitude of +2 V and a duration of 105 ps, while the *Reset* pulse has an amplitude of +3.3 V and a duration of 120 ps. The ON- and OFF-state are evidenced by the sub-ns time response, meaning that the memristor works as a switch up to 20 GHz.

Further developed memristors based on monolayers, such as MoS_2_ or h-BN, rely upon the concepts described above, i.e., the monolayer is embedded in the central conductor, the ON resistance is kept low (at 10–20 Ω), and the results show that the monolayer memristors are very good microwave switches, having low insertion loss (<2 dB) and high isolation (between −25 and −30 dB); moreover, they are able of high-power handling [9,10]. The first experimental results show that these memristors are filament memristors, even if they are monolayers. 

## 3. Atomic-Scale Nano-Ionic Memristor Microwave Switches

There exist other types of memristors which are good microwave switches, such as nano-ionic memristors [11]. Cations, anions or a combination of both can be used for the implementation of such memristors. The implementation via cations is simple and effective, as it is based on electrochemical metallization. Two separate metallic contacts, deposited on a dielectric such as SiO_2_, are needed. The two metals are dissimilar, one of them must be electrochemically active (such as Ag or Cu), and the other one is electrochemically inert (such as noble metals, like Pt, Pd, Au). When a positive DC voltage is applied, the Ag electrode produces Ag^+^ cations which migrate to the inert electrode. In time, metallic filaments are formed between the active and the inert electrodes, thus obtaining a switch in the ON-state. The distance between the electrodes must be in the range 10–40 nm. 

When a negative voltage is applied, the migration direction is reversed and the filament is destroyed; this implies that the switch enters the OFF-state.

**Figure 4:**
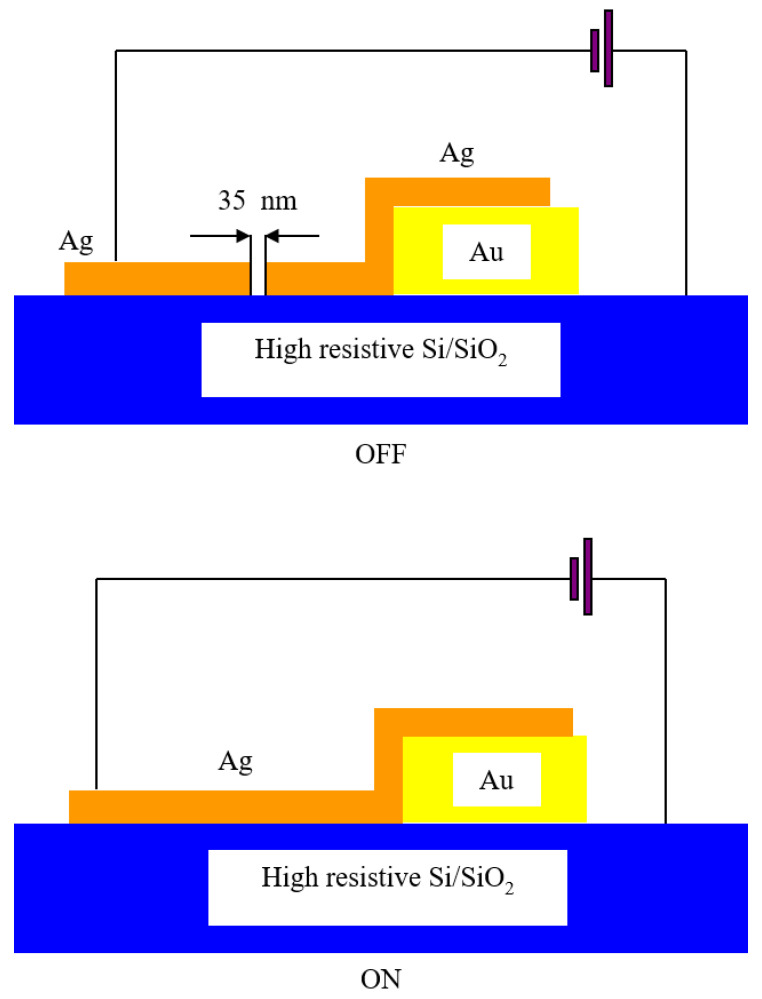
Schematic representation of the nano-ionic memristor microwave switch and its states.

Regarding RF applications, it was shown that Ag cations give rise to filaments that are periodically destroyed between two Au electrodes (which are integrated in the central electrode of a CPW line) when a DC voltage is applied [12]. This way, it was obtained an insertion loss of 0.3 dB and an isolation of 30 dB up to 40 GHz (see Figure 4). The gap between the Ag filaments is 35 nm wide. The compliance current is used to avoid the destruction of the switch in the ON-state.

**Figure 5:**
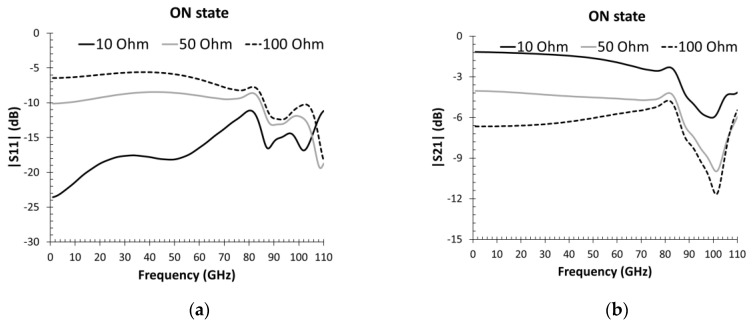
Degradation of memristor microwave switch properties (in ON-state) as a function of different values of *R_ON_*: (**a**) Reflection coefficient |S_11_|; (**b**) transmission coefficient |S_21_| [13].

Besides this, we showed (based on 3D electromagnetic (EM) design–CST Studio Suite^®^ 2014, CST AG, Darmstadt, Germany–integrated with microwave measurements) that the beam of an antenna array can be steered with an angle of ±28° at 2 V bias using the experimental data of either oxide or nano-ionic memristors [13]. A major problem of all these memristor switches is the control of LRS by current compliance, meaning the limitation of current, and this limitation could be different from one memristor to another due to the dynamics of oxygen vacancies and to defects inside the oxide. This could degrade the microwave properties of the memristor switch, as shown in Figure 5.

We see that as soon as *R_ON_* is increased, the degradation of the transmission is clear due to the degradation of the impedance matching. In other words, the switch’s main characteristics degrade visibly if *R_ON_* varies with just an order of magnitude. This effect can be seen in the TiO_2-x_ memristor fabricated and studied by us in [13]. This filament memristor has a low forming (1.8 V) and switching voltages (−1.5 V for *Reset* and 1.2 V for *Set*). The minimum ON resistance of the switch is 42 Ω but, due to the current limitations of electrodes, the minimum programmable ON resistance of the switch is 241 Ω, for a current value of 3 mA. However, other memristors referenced above were able to control the ON resistance at the level of 10 Ω or lower.

We will now present in Table 1 the performances of the above high-frequency memristors extracted from experiments considering the main switch performances in microwaves such as insertion loss, isolation, power handling, but also fabrication performances such as reproducibility denoted as R. We have denoted by V_A_ the voltage necessary to switch in a reversible manner the two states ON and OFF. In the case of memristors two values are resented in the parentheses corresponding with HRS and LRS, respectively.

These atomic-scale microwave switches are compared with RF-MEMS switches which are standard microwave switches today and commercially available. We see that in terms of insertion loss and isolation all the high frequencies switches are comparable, showing good performance. However, the switching voltage for microwave atomically thin memristors is at least tens time lower and their switching time is one order of magnitude lower which is a huge advantage in terms of power consumption and cutoff frequency which is in THz region. Power handling reflects the self-switching of the microwave switches due to applied microwave power. The 2D memristors switches consisting in one single monolayer or having thicknesses up to 4–5 nm support a rather high microwave power of 0.1 W without self-switching phenomena.

The main problem of atomic-thin memristors is their reproducibility since they are fabricated on flakes, and not at the wafer level. The growth of 2D materials is still a challenging problem not solved yet. A graphene monolayer is the single 2D material commercially available on 4-inch or 6-inch Si wafers. The MoS2 or h-BN are commercially available on Si substrates not exceeding 1 cm^2^.

Therefore, we are searching an atomically thin material and a device associated to it which has similar performances with the above atomically-scale switches but is CMOS compatible with high reproducibility. Our answer is ferroelectric tunneling junctions (FTJs) fabricated on HfO2 ferroelectrics which will be described below.

## 4. Atomic-Scale Ferroelectric Junctions as Microwave Switches

The ferroelectric tunneling junctions [14] are MIM-like diodes, where a few-nm-thick ferroelectric is sandwiched between two metal electrodes. An FTJ differs significantly from a MIM diode because the DC current at a certain bias can be switched ON and OFF by an external DC voltage that switches the orientation of the ferroelectric domains. Hence, an FTJ can be seen as a resistive memory with two states, expressed by the so-called tunnel electro-resistance (*TER*) defined as [(*R_ON_* − *R_OFF_*)/*R_OFF_*] × 100% or as *J_ON_*/*J_OFF_*, where *R_ON_* and *R_OFF_* are the resistances of the ON- and OFF- states, while *J_ON_* and *J_OFF_* are the ON and OFF current densities. We have to point out that both definitions are used in the literature, and thus TER is defined either in % or has no units. TER is in the order of 10^2^–10^5^ depending on the ferroelectric type, reaching a giant TER of 6 × 10^6^ in Pt/BaTiO_3_/Nb:SrTiO_3_, which is a metal/ferroelectric/semiconductor FTJ [15]. The resistance, and thus the TER of any FTJ, is dependent on the amplitude of the applied voltage, which is a pulse of few volts with a duration of tens of ns. This dependence has a hysteretic behavior and the FTJ behaves like a memristor due to the dynamics of the ferroelectric domains at various electrical fields [16]. We will consider in the following that the FTJ has attained its maximum TER and we will consider it as a tunneling diode with two states ON and OFF selected by applied DC signals, which are switching the polarity of the built-in field of the ferroelectric material.

FTJ experimental results are reported for many ferroelectrics such as Pt/BTO/NSTO [15], BaTiO_3_/La_0.67_Sr_0.33_MnO_3_ (BTO/LSMO) [17], Sm_0.1_Bi_0.9_FeO_3_/Nb:SrTiO_3_ (SBFO/NSTO) [18]. In this work, we will focus on the FTJ-based on HfZrO ferroelectric, since it is the single CMOS-compatible ferroelectric; furthermore, devices based on such ferroelectrics can be grown at the Si wafer level [19]. The TER effect in HfZrO has been evidenced so far in many experimental research works. A structure made of Pt/Hf_0.5_Zr_0.5_O_2_/Pt has a TER of 20 [20]. A TER higher than 30 was obtained growing HfZrO directly on Si, and using various processes on top TiN electrode [21]. We successfully designed, fabricated and tested a HfZrO-based tunneling diode where the HfZrO having a thickness of 6 nm was grown directly on doped Si [22], showing ON currents higher than 1 mA; after that, we used it in the context of electromagnetic energy harvesting with a TER >10^3^. Decreasing the FTJ thickness down to 1 nm, very recently a TER of 1900% was obtained growing HfZrO directly on Si [23]. In all these examples, the growth technique is atomic layer deposition (ALD). 

Furthermore, the FTJ is an ultrafast switch, since the polarization of the HfZrO thin film makes the ferroelectric domains change their orientation within few ns [24], or even below 1 ns [25], when positive and negative voltages are applied.

**Figure 6:**
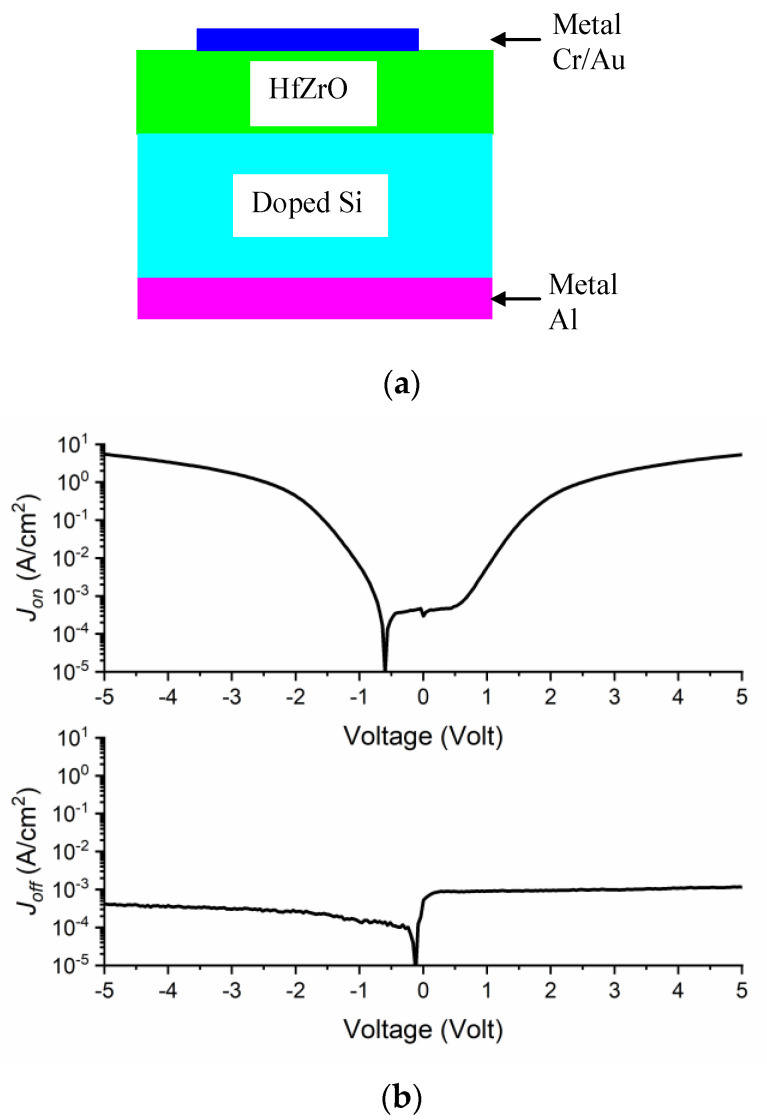
(**a**) Cross-section of the HfZrO-based ferroelectric tunneling junction (FTJ) switch; (**b**) ON (top) and OFF (bottom) current densities as a function of the applied direct current (DC) bias voltage [22].

We used FTJs reported in [22] based on HfZrO (Figure 6a) to demonstrate that an FTJ is acting as a microwave switch. The structural tests, fabrication and measurements are reported in [22]. The FTJ considered henceforth is made up of a 6 nm-thick HfZrO ferroelectric layer grown directly on doped Si substrate, on whose back side an Al bottom electrode with a thickness of 100 nm is deposited. The top electrode is a metal layer of Cr (5 nm)/Au (100 nm), with a contact area of 150 μm × 150 μm. Tens of such FTJ were grown on the same chip and the electrical measurements showed a good yield. The current density is displayed in Figure 6b: here, we can observe two distinct curves in ON- (*J_ON_*) and OFF- (*J_OFF_*) state, as a function of the applied voltage. The dependence is rather symmetric and ambipolar. The ON-state is characterized by a current density of 9 A/cm^2^ at +5 V, while at the same polarization value we have an OFF-current of 1 mA/cm^2^. This high ON-OFF ratio is typical for HfZrO grown directly on Si and is similar with that from [23], where 1 nm-thick HfZrO was grown by ALD directly on Si.

**Figure 7:**
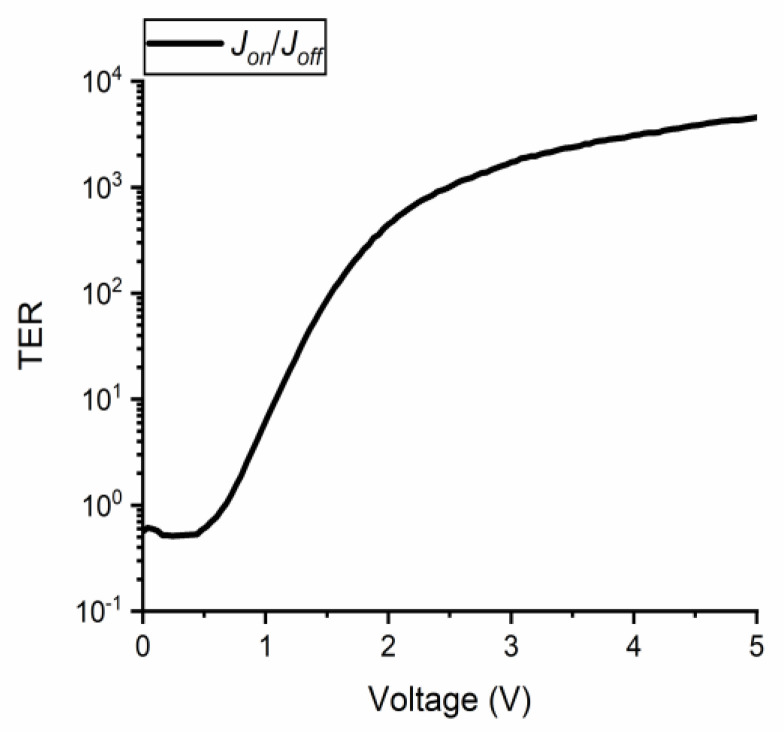
Tunnel electro-resistance (TER) as a function of the applied DC bias voltage.

The TER for positive voltages is displayed in Figure 7 and shows a value of 9 × 10^3^ at +5 V. The ON-state current was obtained by applying a poling ramp signal of +10 V for 20 s, and the OFF-state current was then achieved by using the same poling signal with reversed polarization of −10 V. Repetitive measurements were performed to switch on and off the polarization dipoles and, thus, the ON- and OFF-states of the current, revealing that no significant changes could be observed. Note that when the above FTJs are used in microwaves at the wafer scale, the doped Si (which is the bottom electrode of the FTJs) can be obtained by ion diffusion in selected areas of an HR Si substrate. This is necessary, since doped Si is a lossy substrate for microwaves, while HR Si with a resistivity higher than 10,000 Ω·cm behaves as an insulator.

**Figure 8:**
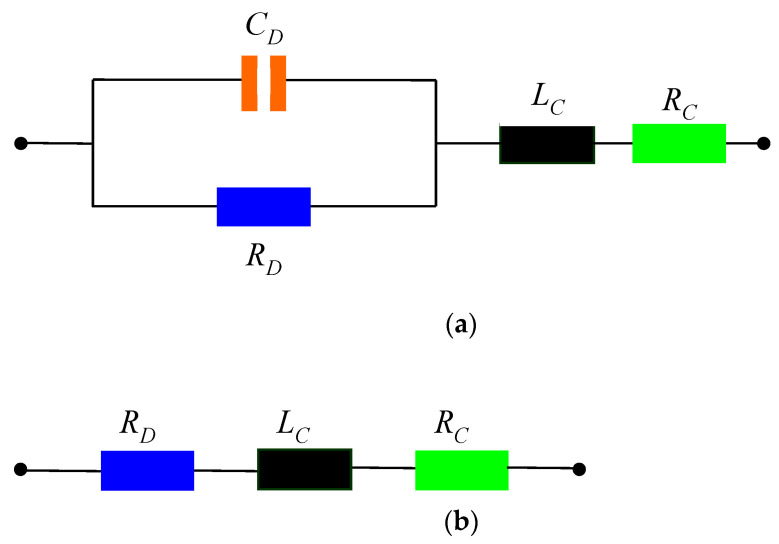
Equivalent circuit of the FTJ-based microwave switch when (**a**) the FTJ is in its reverse polarization state (a negative DC polarity is applied) and (**b**) the FTJ is in its forward polarization state (a positive DC polarity is applied).

The equivalent circuit of the considered FTJ at microwaves is represented in Figure 8. The FTJ is modeled as a parallel resistance-capacitance circuit, where the capacitance *C_D_* is estimated from measurement to be around 200 pF, and the resistance *R_D_* = *∂V*/*∂I* is the differential resistance of the FTJ (since its current-voltage dependence is strongly nonlinear); *R_C_* and *L_C_* are the resistance and inductance of the metal contacts, respectively. We consider that *R_C_* = 0.1 Ω and *L_C_* = 1 nH, as resulted from measurements. We stress here that *C_D_* is the junction capacitance, which plays an effective role only when the diode is in reverse bias (OFF-state, no DC current flowing), while *R_D_* is equal to some tens of MΩ in reverse bias (OFF-state) and to about 2 kΩ in forward bias (ON-state). Keeping this in mind, the microwave behavior of the FTJ-based switch (which is different from its DC behavior) is as follows: in Figure 8a, a negative DC polarity is applied (reverse bias) and the FTJ is in OFF-state in DC and in ON-state at microwaves; vice versa, in Figure 8b, a positive DC polarity is applied (forward bias) and the FTJ is in the OFF-state in DC and in the ON-state in microwaves. In the case of reverse bias, the big capacitance is a short-circuit for microwave signals (since it is equal to an impedance of just 0.08 Ω at 10 GHz), whereas in the case of forward bias, the 2 kΩ resistor behaves as an attenuator, thus blocking the microwaves passing through the FTJ. In other words, the ON-state in microwaves corresponds to the OFF-state in DC and vice versa. In the following, we will avoid any confusion by indicating clearly in the text to whom the ON- and OFF-state are assigned, i.e., to DC or microwave switch. This decoupling between the DC behavior of the FTJ and the functioning of the switch in microwaves has an important advantage regarding the noise. More specifically, as will be shown later on, the FTJ is a low-noise microwave switch.

**Figure 9:**
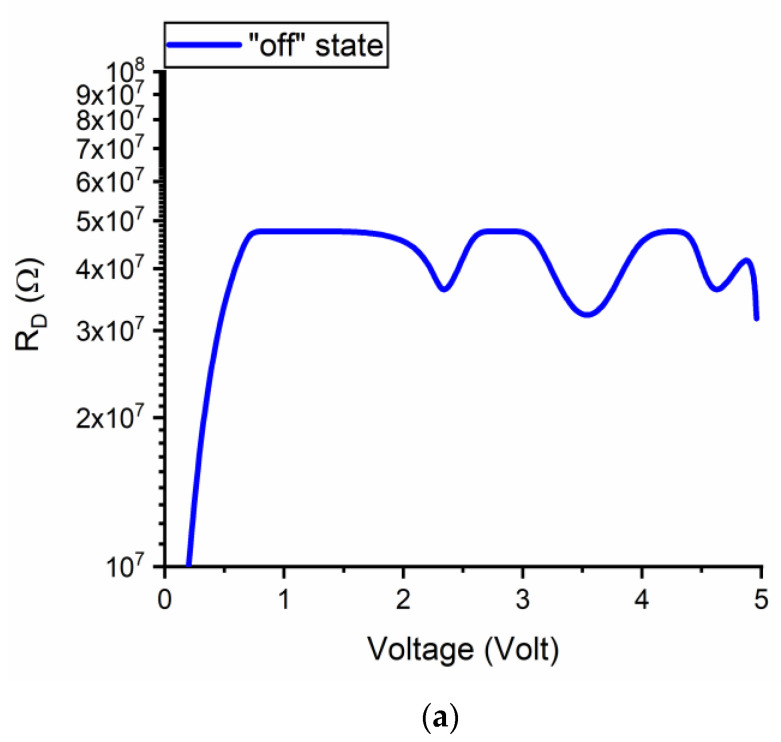

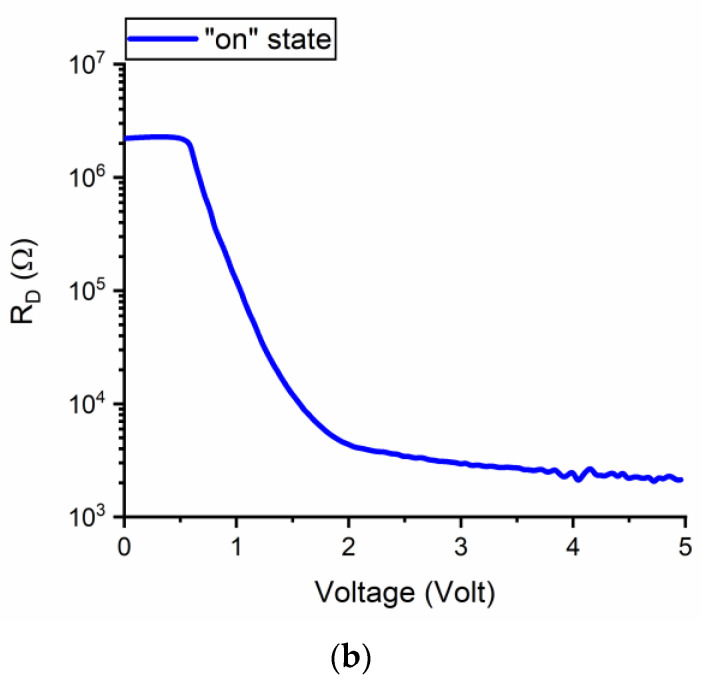
Dependence of the differential resistance *R_D_* on the applied DC voltage for the (**a**) OFF-and (**b**) ON-state of the DC switch.

The dependence of *R_D_* on the applied DC voltage is represented in Figure 9 for the DC OFF-state (Figure 9a) and ON-state (Figure 9b) (ON- and OFF-state of the microwave switch, respectively, as explained above). In Figure 9a, the differential resistance takes values of some MΩ up to about 50 MΩ (as expected), while in Figure 9b it goes down to about 2 kΩ at +5 V.

**Figure 10:**
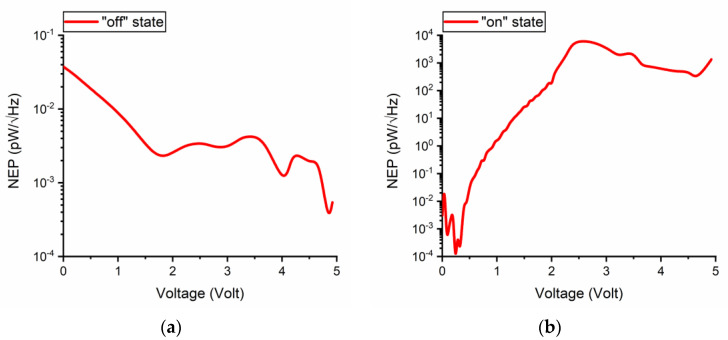
Noise equivalent power (NEP) in the two states of the DC switch: (**a**) OFF (reverse bias) and (**b**) ON (forward bias).

The noise equivalent power (*NEP*) is defined as *NEP* = (4*k_B_TR_D_*)^1/2^/[(*R_D_*^2^/2)(*∂*^2^*I*/*∂V*^2^)] [26] and is represented in Figure 10 in the two states of the DC switch. In the OFF-state, the NEP takes values of some *fW/√Hz*, whereas in the ON-state it reaches about 1 nW/√Hz at +5 V. This means that the microwave switch is a very low-noise device in its ON-state, which represents a major advantage for high-frequency components.

**Figure 11:**
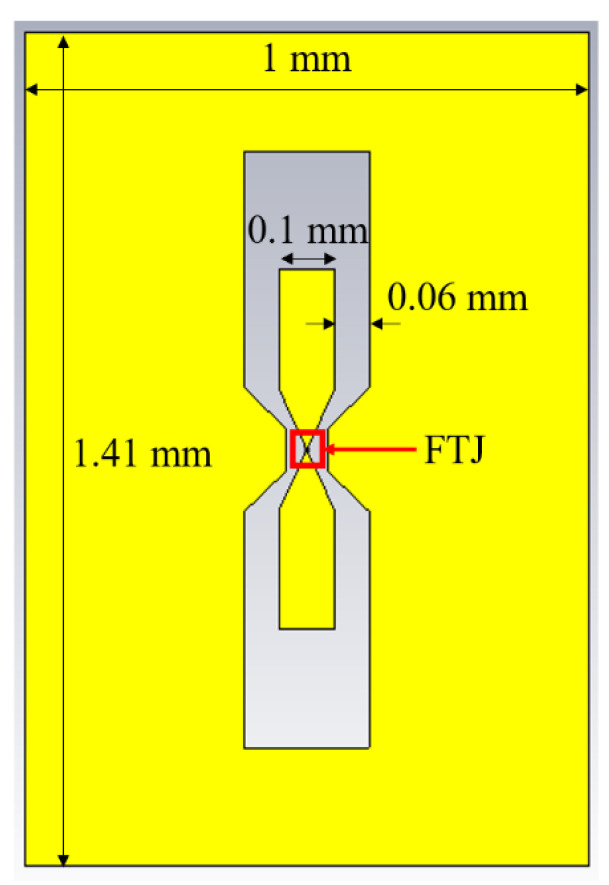
Electromagnetic design of the FTJ-based microwave switch.

**Figure 12:**
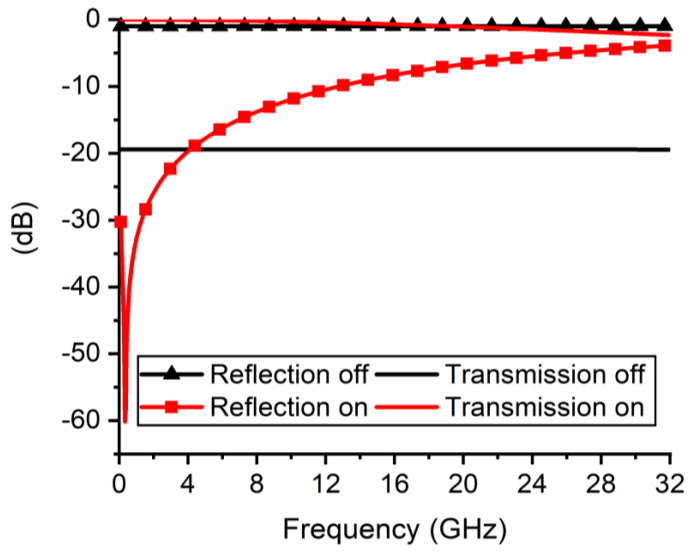
Scattering parameters of the FTJ-based microwave switch in its ON- and OFF-states, in the band 0.1–32 GHz.

In Figure 11 we have depicted the geometry of the FTJ-based microwave switch made on HfZrO/high-resistivity silicon (HR Si) substrate, with the ferroelectric layer having the same thickness as in the previously reported experiments [22]. The simulation results of this FTJ-based microwave switch are displayed in Figure 12. Here we show the scattering parameters, in terms of modulus of the reflection coefficient (|S_11_|) and of the transmission coefficient (|S_21_|), in the ON- and OFF-state of the microwave switch in the band 0.1–32 GHz. In the OFF-state of the microwave switch, |S_11_| is about −0.98 dB over the whole frequency range, whereas |S_21_| is about −19.4 dB. In the ON-state of the microwave switch, in the X band |S_11_| is between −10.22 dB and −13.8 dB (and it remains better than −6 dB up to 22.14 GHz), whereas |S_21_| is between −0.2 dB and −0.44 dB. These results confirm that the FTJ-based microwave switch ensures good performance in a large band in terms of both isolation (|S_21_| in OFF-state) and insertion loss (|S_21_| in ON-state).

Thus, we see that FTJ is a good microwave switch that could have less than 1 ns switching times, low applied DC voltages allowing reversible switching between ON and OFF states, and is highly reproducible using existing clean-room technologies and ALD deposition methods.

**Figure 13:**
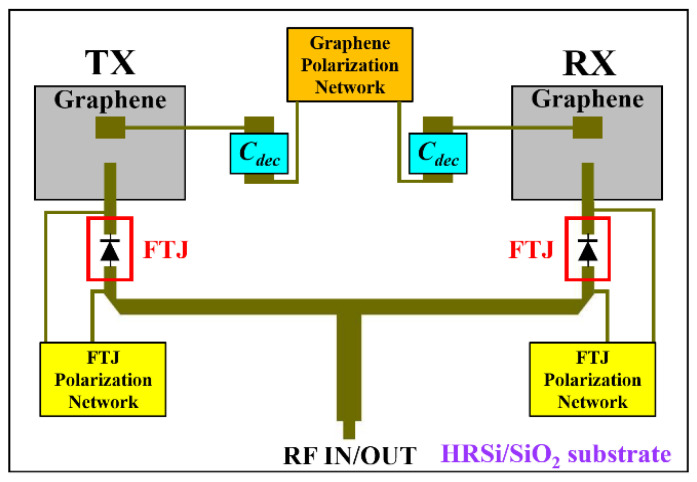
Schematic design of the “smart” transmitter/receiver (T/R) module in the X band, encompassing two FTJ-based microwave switches, two graphene patch antennas and the polarization networks for antennas and switches.

Finally, in Figure 13 we present a transmitter/receiver (T/R) module based on the FTJ-based microwave switch under study. The T/R module consists of a 2-element patch antenna array with operating frequency in the X band. Each antenna is made of a 110-nm-thick graphene multilayer, with overall dimensions of 5.5 mm × 4 mm (width × length), grown on a SiO_2_/HR Si substrate (300 nm/525 μm). The conductivity of the two graphene patches can be ideally tuned by a top-gate configuration using a 30 nm-thick HfO_2_ layer, two decoupling capacitors (*C_dec_*) and a polarization network. This way, we can tune the gain and the operating frequency of each antenna [27,28] thus conferring “smart” characteristics to the T/R module. We simulated the single graphene patch and the array made of two elements using the 3D EM simulator CST Studio Suite^®^, then we used the resulting 1-port (or 2-port) scattering matrix to simulate at the circuit level the entire T/R module (by NI AWR Design Environment^®^, AWR Inc., El Segundo, CA, USA). In detail, each antenna is connected to an FTJ-based microwave switch, and each switch can be biased independently in order to achieve a greater degree of freedom in the control of the two FTJs.

**Figure 14:**
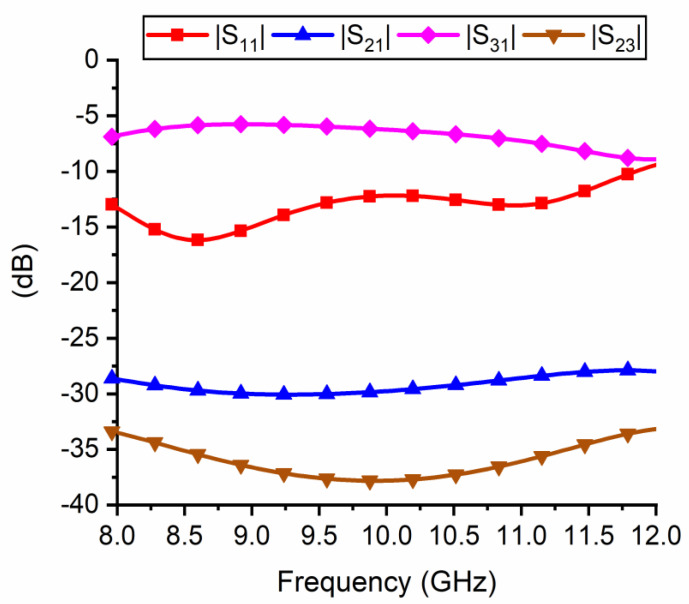
Scattering parameters of the T/R module in TX mode. The three ports have the following significance: port 1 is the “RF IN” port where the RF excitation signal is injected, port 2 is connected to the RX antenna (after the switch “off”), port 3 is connected to the TX antenna (after the switch “on”).

From the circuit simulations, we found that in the X band, when both microwave switches are in their OFF-state, the isolation between them is more than 50 dB. Otherwise, when one of the switches is ON and the other one is OFF, the isolation between them is at least 30 dB. If we consider the T/R module in either transmitting or receiving mode (hence, with a single microwave switch in its ON-state), we observed that the reflection coefficient of the active antenna (as seen from the “RF IN/OUT” port in Figure 13) is better than −10 dB all over the X band, hence the matching to the input impedance of 50 Ω is very good. On the other hand, the power transmission from the “RF IN/OUT” port towards the other antenna is less than −27 dB on the entire X band, hence the antenna connected to the microwave switch in its OFF-state is completed isolated from the excitation port. Furthermore, the isolation between the two antennas is at least 33 dB, hence there is no transmission of microwave power between the two patches, meaning that they are basically decoupled. The results of the simulation are presented in Figure 14. In this figure we study the T/R module in TX mode. In this case, we will consider the following: port 1 is the “RF IN” port where the RF excitation signal is injected, port 2 is connected to the RX antenna (after the switch “off”), and port 3 is connected to the TX antenna (after the switch “on”). Figure 14 shows the scattering parameters which relate these three ports.

The 110 nm-thick graphene multilayer has been already fabricated (Plasma Enhanced Chemical Vapor Deposition (PECVD) NANOFAB 1000, Oxford Instruments, Abingdon, Oxfordshire, UK) and fully characterized (4-point probe, Raman spectroscopy, Atomic Force Microscopy (AFM)), exhibiting a conductivity of about 16,150 S/m and a Root Mean Square (RMS) roughness of 3.6 nm (these data were used to properly simulate the patch antennas). The further processing of the graphene/SiO_2_/HR Si wafer is still on-going for the optimization of the HfO_2_ layer in the top-gate configuration. After that, a complete set of graphene patches and 2-element arrays will be fabricated and tested.

## 5. Conclusions

In this paper, we have presented a brief account of switches for microwaves. Another recent overview of RF switches focused on resistive switching memories is found in [29]. We have shown in this review that the tendency for microwave switches is miniaturization towards an atomic scale, following the same path as transistors having channels made of a 2D material monolayer. Indeed, a single monolayer of h-BN or MoS_2_ is needed for a reliable microwave switch. The main problem is the growth of 2D materials at the wafer scale which is still a difficult issue. Therefore, we have proposed FTJ based on HfO2-based ferroelectrics as a microwave switch which is suitable for low-noise applications in the X band (8–12 GHz). In detail, in the OFF-state the switch provides an isolation of about 20 dB, while in the ON-state the insertion loss is less than 0.5 dB. The unique properties of FTJs allow ultrafast switching times (hundreds of fs) with “smart” reconfigurability characteristics, in the sense that the OFF- and ON-states can be selected by simply reversing the polarity of the poling signal. Finally, an application of practical interest has been proposed, consisting of a 2-element array of graphene patch antennas, featuring gain tunability by applying the proper bias to the graphene layer. Each antenna is connected to an FTJ-based switch, hence creating a “smart” CMOS-compatible T/R module in the X band, able to provide an excellent isolation between the two patches thanks to the ferroelectric microwave switches.

## Figures and Tables

**Table 1:** The microwave performance of atomically thin memristors extracted from experimental data.

Microwave Switch Type	Voltage V_A_ (V)	Insertion Loss (dB)	Isolation Loss (dB)	Bandwdth (GHz)	Switching Time	Power Handing (W)	R
Radio-Frequency Micro-Electro-Mechanical Systems (RF-MEMS)	40–80 V	−0.2–−05	−30–−40	0.1–50	10–20 μs	8–10	high
memristor Ta_2_O_5_	(−3, +2 V)	−1.5	−16	0.1–20	0.12 ns	0.1	high
memristor h-BN monolayer	(−1, +1.5)	−0.25	−40	0.1–50	10 ns	0.1	low
memristor MoS_2_ monolayer	(−1, +1)	−0.4	−20	0.1–50	<30 ns	0.01	low
memristor nanoionic	(0, 0.4)	−0.2	−35	10–40	<20 ns	0.1	low

## Data Availability

The data presented in this study are available on request from the corresponding author. The data are not publicly available due to intellectual property.
